# Case report: the challenges of psychogenic polydipsia

**DOI:** 10.1093/omcr/omaf278

**Published:** 2025-12-26

**Authors:** Anirban Raha, Aparna Mordekar

**Affiliations:** Older Peoples Liaison Psychiatry Team, The Longley Centre, Norwood Grange Drive, Sheffield, S5 7JT, Sheffield Health Partnership University NHS Foundation Trust, UK; Older Peoples Liaison Psychiatry Team, The Longley Centre, Norwood Grange Drive, Sheffield, S5 7JT, Sheffield Health Partnership University NHS Foundation Trust, UK

**Keywords:** Paranoid Schizophrenia, Psychogenic Polydipsia, Hyponatraemia Naltrexone

## Abstract

**Aim:**

We describe a case report of a 71-year-old with a long history of Paranoid Schizophrenia presenting with psychogenic polydipsia.

**Background:**

Psychogenic polydipsia is a rare and challenging complication of Paranoid Schizophrenia. Research is limited with regards to how to treat this effectively, however the management normally involves fluid restriction and behavioural therapy.

**Method:**

We looked through his history and liaised with multiple specialities to compile this report.

**Result:**

This patient has long history of psychogenic polydipsia. More recently he presented with his chronic hyponatremia. To aid reduction of his hyponatraemia, his duloxetine was stopped, and his olanzapine dose reduced.

**Conclusions:**

There is evidence in the role of naltrexone in reducing compulsive drinking. We trialled this at 25 mg and the patient’s drinking behaviour has reduced in the community.

## Introduction

The normal recommended intake of water for an adult is 2–4 litres per day. Polydipsia is defined as having an excess of 6 litres of water per day [1]. Psychogenic polydipsia is an uncommon complication of neuropsychiatric conditions, particularly schizophrenia. It occurs in 6%–20% of psychiatric patients [2]. Psychogenic polydipsia is characterised by excessive water drinking in the absence of a physiological stimulus. A consequence of psychogenic polydipsia is hyponatraemia, which is characterised by sodium levels below 134 mmol/l. Mild hyponatraemia is between 134 mmol/l and 130 mmol/l; 125–129 mmol/l is moderate, and 125 mmol/l or below is deemed severe hyponatraemia [1]. Symptoms usually appear in severe hyponatraemia, and can include lethargy, anorexia, agitation, disorientation, seizures and coma [3].

Various theories hypothesise the aetiology of psychogenic polydipsia. These include positive symptoms of schizophrenia, stress reduction and efforts to counteract the side effects of anticholinergic medications. Increased dopamine levels are thought to stimulate thirst centres, and it has been suggested that dopaminergic super sensitivity may explain why psychogenic polydipsia often occurs in the later stages of schizophrenia after years of exposure to typical neuroleptics [2].

We present the case of a patient with an established diagnosis of paranoid schizophrenia, and the challenges of managing his psychogenic polydipsia.

## Case report

This case report describes a 71-year-old male with a background of Paranoid Schizophrenia, who had been seen on several occasions by the Older Peoples Liaison Psychiatry Team in Sheffield. He had an extensive history with the community mental health team; Initially presenting with depression and anxiety in 2006, then developing psychotic symptoms in 2009 resulting in a psychiatric inpatient admission and a diagnosis of Paranoid Schizophrenia. Following this, there was a period of stability and had been discharged from the team in recent years. His medical history included hypertension, hypercholesterolaemia and mild aortic regurgitation.

He was initially brought to our attention regarding his polydipsia in May 2023, when he was admitted due to a history of confusion and dizziness. The patient exhibited a history of compulsive water consumption, resulting in significant hyponatremia with sodium levels of 121 mmol/L (See Table 1 for all initial Biochemistry). Management included fluid restriction and the prescription of artificial saliva spray, which provided temporary improvement before discharge.

**Table 1:** Biochemistry summary from each admission.

Admission Date	Sodium (mmol/L) 133–146 mmol/l	eGFR (ml/min/1.73m^2^)	Serum Osmolality (mOsm/kg) 275–295 mOsm/kg	Urine Sodium (mmol/L) 40–200 mmol/L	Notes
24/04/2023	121	90	252		First admission; fluid restriction
13/09/2023	130	90			
30/09/2023	117	90		53	Lowest result. Duloxetine stopped
12/10/2023	124	84	250		
30/10/2023	117	86			Found on floor confused
05/11/2023	124	84	261		Olanzapine reduced
30/11/2023	122	84	260		
04/12/2023	127	90			
23/12/2023	122	90			Commenced Naltrexone
09/01/2024	128	90			
06/02/2024	135	87			Stable on Naltrexone

He had several readmissions between May and June 2023 for general malaise and abdominal pain, attributed to recurrent hyponatremia and was managed with fluid restriction.

His recurrent admissions intensified in September 2023. Following a 10-day admission, during which his sodium dropped to 127 mmol/L, he was repeatedly reviewed by the Older Peoples Liaison Psychiatry Team. The patient exhibited a strong compulsion to drink, reporting he ‘could not stop drinking,’ and illustrated the severity of this by locking himself in a bathroom to drink from the sink despite being on fluid restriction. Crucially, he retained insight, acknowledging the risk of death, and displayed no active psychotic or affective symptoms. His current psychotropic medications were duloxetine 60 mg OD, olanzapine 20 mg OD and zopiclone 7.5 mg PRN.

The subsequent two readmissions (one resulting in a month-long inpatient stay) quickly followed, with his hyponatremia worsening to 117 mmol/L and presenting with confusion. During this prolonged admission, a medication review was undertaken, and the decision was made to stop Duloxetine due to its potential impact on his sodium levels. A concurrent referral was made to the Older Adult Community Mental Health Team (CMHT) to explore psychological interventions, including Cognitive Behavioural Therapy (CBT), to mitigate the compulsive drinking. The CMHT noted that Cognitive Behavioural Therapy (CBT) had been exhausted in the past and formulated that the patient was likely seeking containment in a safe environment away from home stressors. During the prolonged admission, the medical team stopped his amlodipine. Following a period of fluid restriction (1.5 L), his sodium levels improved to 130 mmol/L, and he was discharged. However, this stability was short-lived, leading to two rapid readmissions in early November 2023. During the first admission, he presented highly confused with a significant drop in sodium to 117 mmol/L, requiring treatment with hypertonic saline. During the subsequent admission, his olanzapine dose was reduced from 20 mg to 15 mg (due to known links between antipsychotics and hyponatremia [4]). At this time, he was noted to be drinking more than 100 cups of water per day, with the Liaison Psychiatry Team observing repetitive speech, though he maintained insight into his excessive water intake.

There were questions from the medical ward regarding whether he needed detention in the psychiatric unit. However, he did not display any psychotic features, had insight into his presentation and wanted to work with the mental health team in the community. The patient did not warrant a mental health admission. We agreed to try our supported discharge team again to determine if they could provide distraction techniques. We also liaised with our pharmacy team to ascertain any medications that could help with his compulsive drinking behaviour. Some studies have indicated that bupropion [5] and natlrextone [6] have some benefits with compulsive drinking associated with psychogenic polydipsia. We considered potentially trialling naltrexone owing to the lack of availability of bupropion in our area; however, if used, it would be off licence.

We arranged a professional meeting between members of the liaison psychiatry team, older adult community mental health team, and the patient’s general practitioner. The psychologist from the CMHT highlighted how the patient would not be appropriate for complex CBT, especially as the last few reviews indicated possible cognitive impairment. The CMHT, however, agreed to form a clinical formulation to help support staff working with the patient to understand his presentation. It would also be useful to explore family dynamics, as in the past, this has been a causal factor for admission to hospital, and getting away from his home life. After the professional’s meeting, the pharmacological plan was discussed with his daughter, as patient lacked capacity, and the daughter had lasting power of attorney (LPA). She agreed to this. The CMHT also agreed that his cognition would need to be formally assessed. We discussed the option of trialling naltrexone, with the GP agreeing to commence it at 25 mg OD. The CMHT would then review this and increase it to 50 mg after a 6-week trial.

By December 2023, the patient was admitted to the hospital three more times due to his hyponatraemia. During this period, he underwent an MRI scan, which indicated a generalised cerebral atrophy that was advanced for his age, with slight disproportionate atrophy in the frontal and temporal lobes. There was also a small old established infarct in the right centrum semiovale.

**Figure 1:**
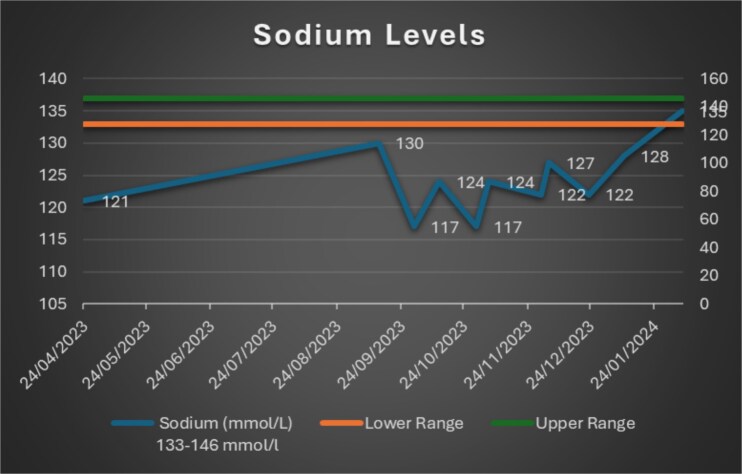
Serum sodium results graph (reference range: 133–146 mmol/l).

Following a recent admission in February 2024 (due to a social admission), he was transferred to a care home to determine what additional needs he may have. At the time of writing, his sodium had remained within the normal limits (see Figure 1 indicating trend of serum sodium levels). After the CMHT review, naltrexone was continued at 25 mg, given the improvement.

Since his transfer into a care home, he has remained stable. It has been reported that he does seek out more water for drinking but is easily able to be redirected. The plan from the CMHT was to do a neuropsychological assessment to aid in the diagnostic process, however due to the change his living circumstances, they felt that a cognitive assessment would not be appropriate and could have caused unnecessary burden.

## Discussion

Psychogenic polydipsia is associated with several conditions, including schizophrenia, bipolar disorder, and psychotic depression. However, it is most commonly associated with schizophrenia, and some studies suggest that 11–20% of patients with schizophrenia have associated psychogenic polydipsia, and among these, approximately 20% develop hyonatreamia [7]. The pathogenesis of polydipsia is unclear; however, several theories have been proposed. One theory postulates hypersensitivity to vasopressin, an increase in dopamine activity and a defect ion osmoregulation. Another theory suggests that stimulation of thirst centres by elevated dopamine levels and drinking to counteract the anticholinergic side effects of psychotropic medications [8].

Differential diagnoses (See Table 2) include Diabetes Insipidus, and other secondary causes of psychogenic polydipsia: Syndrome of Inappropriate Antidiuretic Hormone Secretion (SIADH) and Diabetes Mellitus types 1 and 2. Primary polydipsia is caused by excessive fluid intake without an underlying medical cause, while secondary polydipsia can be medication-induced (i.e. from diuretics or laxatives) or due to a medical condition. Diabetes insipidus is characterised by polydipsia, polyuria and diluted urine, and is diagnosed by a water deprivation test, vasopressin test and MRI scan to check for damage to the hypothalamus. SIADH is characterised by tremors, muscle cramps, nausea, depression, seizures, memory deficits, vomiting and confusion. This condition is usually diagnosed by urinalysis and osmolality tests. Diabetes Mellitus is characterised by fatigue, polyuria, polydipsia, weight loss (type 1) or weight gain (type 2). This is usually diagnosed by urinalysis and measurement of blood glucose and HBA1c^1^. Regarding psychogenic polydipsia, other differentials need to be ruled out [3]. In this case, all medical causes were excluded. As mentioned previously, symptoms usually appear in severe hyponatraemia (below 125 mmol/l), which include lethargy, anorexia, agitation, disorientation, seizures and coma.

**Table 2:** Differential diagnosis.

Diagnosis	Primary Problem	Symptoms	Diagnostic Criteria	Management
Psychogenic Polydipsia	Excessive fluid intake	Excessive water intake despite body not needing it. Behavioural	Underlying psychiatric illness, serum electrolytes and/or urine osmolality test	Fluid restriction
Diabetes Insipidus	Reduced ADH	Polyuria, polydipsia and diluted urine	Serum electrolytes, water deprivation test, vasopressin test, MRI (detect damage/abnormality hypothalamus)	Desmopressin
Diabetes Mellitus	Hyperglycaemia	Polyuria, polydipsia, fatigue, weigh gain and loss, repeated infections.	Fasting blood glucose, HbA1c, Urinalysis.	Diabetic medications
SIADH	Increased ADH	Tremor, muscle cramps, nausea, confusion, seizures.	Serum electrolytes, urine and serum osmolality, toxicology and Thyroid hormone levels.	Fluid restriction, stop causative medication, surgery

There are no specific guidelines for the treatment of psychogenic polydipsia. However, there are protocols for the treatment of the symptoms. When presenting with acute hyponatraemia in secondary care with moderate to severe symptoms, the management is to treat with hypertonic saline (to prevent cerebral oedema). For patients with hypervolaemia, fluid restriction is recommended to prevent fluid overload. Fluid restriction is also recommended when no clear cause of SIADH is found following investigations [9].

Behavioural cognitive therapy is the mainstay of treatment for psychogenic polydipsia. Therapists used cognitive techniques to address thoughts leading to drinking behaviour and then implemented a behavioural program to restrict water intake. They implemented a stimulus control device (i.e. a 500 mL water jug was used as a stimulus control device and the patient was instructed to fill it only six times daily to achieve a goal of less than 3 L for water restriction), and coping skills (substituting ice cubes for drinks, taking small sips, distracting activities) [2]. In this case, due to the patients declining cognition, he would not have been able to engage in therapy.

There are no specific protocols with regard to drug treatments for psychogenic polydipsia, and the only evidence available comes from case reports and small clinical trials, and larger trials need to be conducted. Atypical antipsychotics have been shown in case reports to alleviate symptoms (low dose risperidone and olanzapine), and clozapine has been shown to be beneficial in a small trial. This could be due to dopamine receptor regeneration following chronic antipsychotic neuroleptic administration. It is theorised that this reduction in dopamine sensitivity decreases thirst stimulation [2].

During a literature search, we found a small clinical trial that had been performed with naltrexone. Naltrexone is an opioid receptor antagonist. This prevents dopaminergic activity after alcohol consumption and reduces its reward effects. It is used to aid in abstinence from opioid and alcohol misuse [10]. It has been theorised that naltrexone can play a significant role in improving self-drinking behaviour. This is correlated by the idea of the links between polydipsia on opiate receptors and addictive behaviour [6]. Some papers describe observations of stereotypic behaviours in patients with psychogenic polydipsia. They share similarities with the behaviours observed in Lesch-Nyan syndrome, where they show diminished symptoms after treatment with opiate antagonists [6].

In an open design study, naltrexone 50 mg OD was added for a period of 6 weeks to the anti-psychotic regime of seven inpatients with psychogenic polydipsia. These patients showed a significant reduction in compulsive drinking behaviour during their last treatment period. However, the results of the study were limited due to the small sample size and warrant larger studies to support the hypothesis that behaviours [6].

## Conclusion

This case highlights the challenges of managing psychogenic polydipsia and that there are no definitive treatments. In light of his MRI findings, he will need further assessment for underlying cognitive deficits, as schizophrenia can lead to neurocognitive decline in old age.

To our knowledge, there have not been any documented reports on the use of naltrexone in the management of psychogenic polydipsia in older adults with schizophrenia; only one small clinical trial was identified linking naltrexone to managing psychogenic polydipsia generally. The uniqueness of this case lies in the patient’s history of repeated admissions with severe hyponatraemia secondary to psychogenic polydipsia, the off-license use of naltrexone, and the eventual stabilisation achieved in a supported care home environment through multidisciplinary collaboration. This highlights a potential role for opioid antagonists such as naltrexone as an adjunctive treatment in cases where standard behavioural and pharmacological strategies have failed or are not feasible.
